# T2-weighted MRI-based radiomics for discriminating between benign and borderline epithelial ovarian tumors: a multicenter study

**DOI:** 10.1186/s13244-022-01264-x

**Published:** 2022-08-09

**Authors:** Mingxiang Wei, Yu Zhang, Genji Bai, Cong Ding, Haimin Xu, Yao Dai, Shuangqing Chen, Hong Wang

**Affiliations:** 1 Department of Radiology, The Affiliated Suzhou Hospital of Nanjing Medical University, Suzhou, Jiangsu China; 2 Gusu School, Nanjing Medical University, Suzhou, Jiangsu China; 3 Department of Radiology, Dushu Lake Hospital Affiliated to Soochow University, Suzhou, Jiangsu China; 4 Department of Radiology, The Affiliated Huaian No. 1 People’s Hospital of Nanjing Medical University, Huaian, Jiangsu China; 5 Department of Radiology, The First Affiliated Hospital of Soochow University, Suzhou, Jiangsu China

**Keywords:** Ovary, Radiomics, Machine learning, Magnetic resonance imaging

## Abstract

**Background:**

Preoperative differentiation between benign and borderline epithelial ovarian tumors (EOTs) is challenging and can significantly impact clinical decision making. The purpose was to investigate whether radiomics based on T2-weighted MRI can discriminate between benign and borderline EOTs preoperatively.

**Methods:**

A total of 417 patients (309, 78, and 30 samples in the training and internal and external validation sets) with pathologically proven benign and borderline EOTs were included in this multicenter study. In total, 1130 radiomics features were extracted from manually delineated tumor volumes of interest on images. The following three different models were constructed and evaluated: radiomics features only (radiomics model); clinical and radiological characteristics only (clinic-radiological model); and a combination of them all (combined model). The diagnostic performances of models were assessed using receiver operating characteristic (ROC) analysis, and area under the ROC curves (AUCs) were compared using the DeLong test.

**Results:**

The best machine learning algorithm to distinguish borderline from benign EOTs was the logistic regression. The combined model achieved the best performance in discriminating between benign and borderline EOTs, with an AUC of 0.86 ± 0.07. The radiomics model showed a moderate AUC of 0.82 ± 0.07, outperforming the clinic-radiological model (AUC of 0.79 ± 0.06). In the external validation set, the combined model performed significantly better than the clinic-radiological model (AUCs of 0.86 vs. 0.63, *p* = 0.021 [DeLong test]).

**Conclusions:**

Radiomics, based on T2-weighted MRI, can provide critical diagnostic information for discriminating between benign and borderline EOTs, thus having the potential to aid personalized treatment options.

**Supplementary Information:**

The online version contains supplementary material available at 10.1186/s13244-022-01264-x.

## Key points

- T2-weighted MRI-based radiomics could preoperatively discriminate benign and borderline EOTs.
- Radiomics combined with clinical/radiological characteristics help differentiate benign and borderline EOTs.
- Different machine learning algorithms had different diagnostic performances.

## Background

Borderline epithelial ovarian tumors (EOTs) have been classified as a separate category and account for approximately 10% to 20% of all EOTs [1, 2]. The peak incidence of borderline EOTs was at the age of 55–59 years, with a rate of approximately 4.5–7.3/10,0000 [3, 4]. The median age at diagnosis for borderline EOTs was around 50 years, and more than a third of cases occurred in women younger than 40 years of age [3–5]. One study showed that patients with borderline EOTs were mostly asymptomatic or had only abdominal pain or menstrual abnormalities, and borderline EOTs are closer to benign ones than to malignant ones [6]. Pathologically, borderline EOTs have no destructive stromal invasion [7]. However, borderline EOTs are considered as precursor lesions to ovarian cancer of the corresponding histologic type [8]. Misdiagnosing borderline EOTs as benign lesions may result in some patients choosing nonsurgical treatment, such as ultrasound follow-up, which carries a risk of resulting in borderline EOTs malignant transformation or spread [9]. Although conservative fertility surgery is recommended for both benign and borderline EOTs, gynecologists require a more objective and reliable preoperative evaluation for the latter to weigh the stakes between tumor recurrence and fertility preservation [10, 11]. Hence, there is a need for accurate preoperative differentiation between borderline and benign EOTs.

Ultrasound is the first-line choice for ovarian tumor diagnosis, but its sensitivity for the diagnosis of borderline EOTs was only 0.660 [12]. Magnetic resonance imaging (MRI) has been demonstrated to be superior to ultrasound for assessing complex ovarian lesions [13]. The advent of the ovarian-adnexal reporting data system (O-RADS) MRI score has improved the diagnostic accuracy of ovarian neoplasms [14]. Nevertheless, studies have shown that differentiating borderline from benign EOTs on conventional MRI remains a nontrivial challenge for radiologists due to the complexity of the tumor morphology and the similarity of the MRI signs [15, 16]. For example, Park et al. reported that there is no significant difference between borderline and benign EOTs in the number and size of separations on conventional MRI [17]. Upon considering the limitations of conventional MRI in differentiating borderline EOTs from benign EOTs, there is a clear need for new methods to aid the diagnosis.

Radiomics has emerged as a powerful tool in oncology research. This method can convert routine medical images into quantitative features, thus reflecting valuable information that cannot be identified by the naked eye [18, 19]. To date, MRI-based radiomics methods have been shown to assist radiologists in differentiating between type Ι and type ΙΙ epithelial ovarian cancers [20] and classifying benign and malignant ovarian tumors [21, 22]. A phantom study demonstrated that T2-weighted (T2W) images have advantages in radiomics because interobserver reproducibility was better for radiomics features derived from T1-weighted images (intraclass correlation coefficient [ICC] ≥ 0.75) than from T1-weighted images (ICC = 0.60–0.71) [23]. Previous studies have focused on the differentiation between malignant and borderline EOTs [24, 25]. However, to our knowledge, the potential role of T2W MRI-based radiomics in differentiating between benign and borderline EOTs has not been established.

Therefore, the primary aim of this retrospective multicenter study was to evaluate T2W MRI-based radiomics in preoperatively distinguishing between benign and borderline EOTs.

## Methods

### Patients

**Fig. 1:**
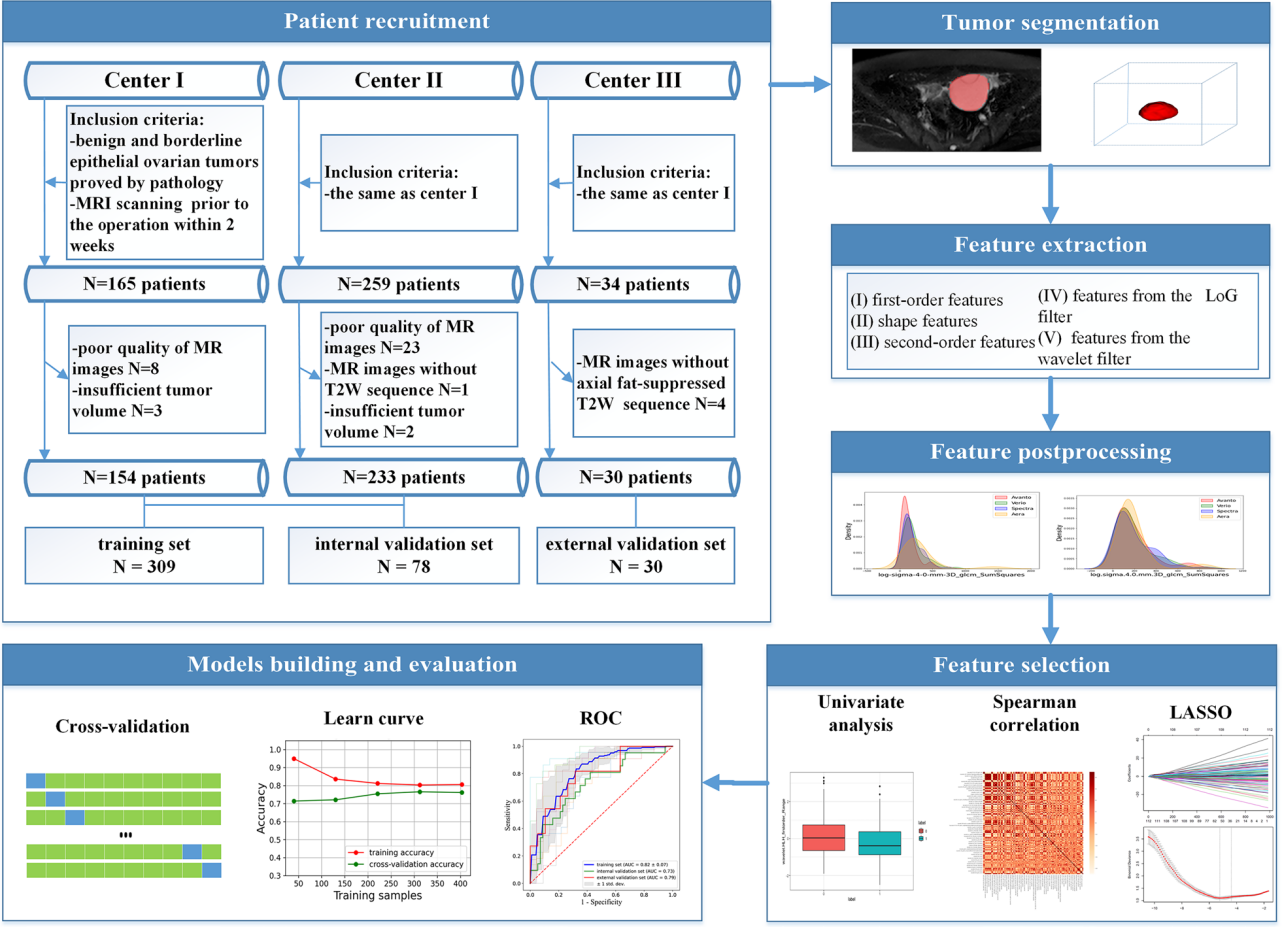
Patient recruitment and workflow of radiomics analysis

This retrospective multicenter study was approved by the institutional review boards, and the written informed consent was waived for this study. A review of clinical databases and the picture archiving and communication system was performed to retrospectively enroll consecutive patients from July 2013 to July 2021 at Center I, from January 2016 to June 2021 at Center II, and from January 2017 to January 2018 at Center III. The center information is shown in the Additional file 1 (Section 1). The inclusion criteria were as follows: (1) pathologically confirmed benign or borderline EOTs; (2) MRI scanning performed within at least 2 weeks before surgery. Patients were excluded based on the following criteria: (1) receiving any treatment before MRI examination and biopsy, including chemotherapy or radiotherapy; (2) lack of T2W sequences; (3) poor-quality MR images due to artifacts; (4) the tumor could not be fully displayed because of insufficient volume or too large of a tumor volume. In total, 417 patients (154 from Center I, 233 from Center II, and 30 from Center III) were enrolled in the study. Patients from Center I and Center II were stratified into the training and internal validation sets at a ratio of 8:2. Data from Center III were reserved for external validation set to evaluate the generalizability of the created models to data from separate institutions. The clinical characteristics of all patients, including age, menopausal status, parity, abdominal symptoms, carbohydrate antigen 125 (CA125), and human epididymis protein 4 (HE4), were obtained from patients’ electronic medical records. The details of the recruitment process are shown in Fig. 1.

### Image acquisition and tumor segmentation

Patients scanned on various 1.5 T or 3.0 T units with phased-array coils were all included in this study. Fat-suppressed (FS) T2W images were used in this study. The scanners and imaging parameters of FS T2W are summarized in the Supplementary Materials (Additional file 1: Table S1).

**Fig. 2:**
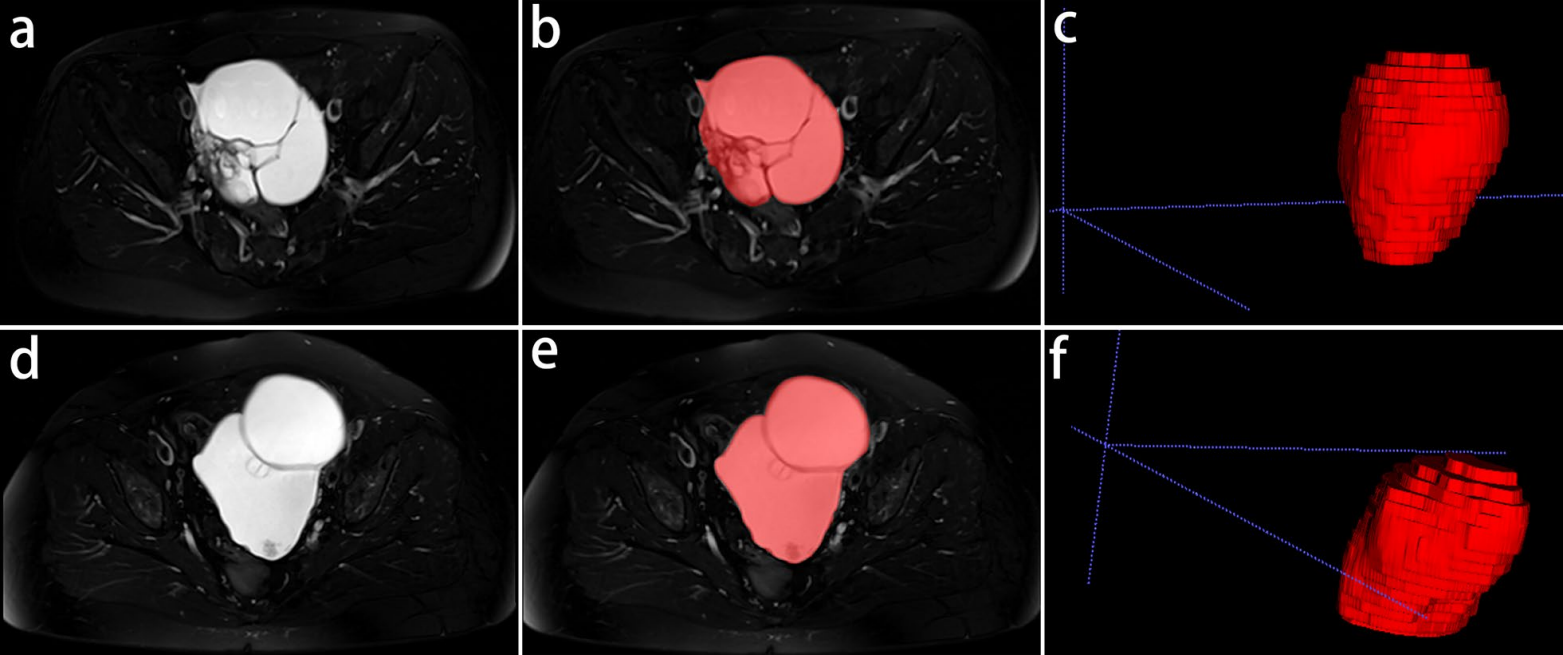
Two representative examples of volumes of interest (VOIs) segmentation of benign and borderline EOTs in axial fat-suppressed T2-weighted images. **a**–**c** A 50-year-old woman with a right benign mucinous cystadenoma; **d**–**f** a 60-year-old woman with a left borderline mucinous cystadenoma; **a**, **d** the original images; **b**, **e** the VOIs of ovarian masses showing in red; **c**, **f** 3D renderings of the VOIs

Tumor volumes of interest (VOIs) containing both the cystic and solid components were manually delineated slice-by-slice on FS T2W images by using ITK-SNAP software (v. 3.8.0, http://www.itksnap.org) [26]. Only the one with the largest maximum diameter on axial images was selected for segmentation if the tumor was multifocal in nature. Two examples of VOIs segmentation are shown in Fig. 2. Radiologist A, who had 10 years of experience in pelvic MRI diagnosis, first segmented the VOIs for all subjects. To evaluate the interobserver reproducibility, the VOIs of 30 patients randomly chosen from the training set were segmented by another radiologist (Radiologist B) who had 5 years of experience in pelvic MRI diagnosis. To assess the intraobserver reproducibility, Radiologist A repeated the segmentation procedure for all patients after one month. The interobserver and intraobserver reproducibility of VOIs was evaluated by ICCs, and ICCs > 0.80 are considered robust and reproducible [27]. The first segmentation of Radiologist A was used to create models. Referring to one previous study [28], the two radiologists who performed VOIs delineation also independently assessed the following conventional MRI characteristics: (1) ascites, which was classified as none, mild﻿ (limited to the Douglas pouch), moderate (limited to the pelvic cavity), or massive (beyond the pelvic cavity); (2) margin, which was classified as well-defined or ill-defined; (3) the number of loculi, which was classified as mild (< 3) or multilocular ( ≥ 3); (4) signal intensity (SI) of the solid component on FS T2W images (compared with adjacent external myometrium), which was classified as none, low, high, or mixed; (5) SI of the cystic component on FS T2W images (compared with urinary bladder), which was classified as low, moderate, or high; and (6) the maximum diameter. Disagreements between the two radiologists were rereviewed in consensus. Some examples referred to the evaluation of signal intensity are shown in Supplementary Materials (Additional file 1: Figs. S1 and S2). The two radiologists were blinded to the histopathologic results and clinical information of the tumors when reviewing MRI images.

### Feature extraction

Before radiomics processing, normalization was used to transform arbitrary gray intensity values into a standardized intensity range; all T2W images and masks were then isotopically resampled to 3 × 3 × 3 mm^3^ by using B-spline interpolation. A total of 1130 radiomics features were extracted from VOIs by using the PyRadiomics package (http://www.radiomics.io/pyradiomics.html) [29] in Python. Most radiomics features follow the image biomarker standardization initiative (IBSI) [30]. The custom settings and detailed information on radiomics features are included in Additional file 1 (Section 2).

### Feature postprocessing

ComBat harmonization was performed on the radiomics features, ﻿which is desirable before building models, as ﻿it reduces the bias caused by different scanners (Additional file 1 [Section 3]) [31–33]. Radiomics features after ComBat harmonization were standardized by *Z*-score normalization (removing the mean and scaling to unit variance). Furthermore, we applied the synthetic minority oversampling technique (SMOTE) in the training set to reduce the bias of the sample imbalance [34]. Finally, features with ICCs < 0.8 were excluded.

### Feature selection

Radiomics features had high dimensionality; thus, several feature selection steps were used. The Mann–Whitney *U* test was first performed to select statistically significant features between benign and borderline EOTs in the training set. Second, the importance weight of each feature was calculated by using Random Forest (RF) algorithm, and the correlation coefficient between every two features was calculated by Spearman correlation analysis. For any pair of features with correlation coefficients > 0.90, the one with the lowest importance weight was removed from the training data. Finally, the Least Absolute Shrinkage and Selection Operator (LASSO) algorithm was used to solve the multicollinearity problem, by selecting diagnosis-related features with only nonzero coefficients. More information on feature selection is shown in Additional file 1 (Section 4 and Additional file 1: Fig. S3).

### Models building and evaluation

We used four different machine learning algorithms to construct radiomics models, including logistic regression (LR), support vector machine (SVM), RF, and Naive Bayes (NB). The best machine learning algorithm was selected by analyzing the fitting and generalization performance. We applied the learning curve to assess the trend of the training and cross-validation scores with the increase in sample size. If both the validation and training scores converge to a stable value, the model is considered not to benefit from additional training data. Furthermore, the learning curve can be used for comparison among multiple models. The higher the training and cross-validation scores, the better the fitting performance; the smaller the gap between the training and cross-validation score, the better the generalizability.

Next, we incorporated the clinical and conventional MRI characteristics that were statistically significant after univariate analysis into the radiomics model (combined model) to explore whether this can further improve the performance. These clinical and conventional MRI characteristics were also fed into a separate model (clinic-radiological model). Multiclass variables in this study were one-hot encoded before model building. To increase the comparability of the models, we selected the same machine learning algorithm as the one used in the best radiomics model. The outcomes of these three models were comprehensively compared to explore the optimal model with the best diagnostic efficiency.

The area under the ROC curve (AUC) was used as the main indicator for model evaluation and comparison. The sensitivity, specificity, positive predictive value (PPV), negative predictive value (NPV), and accuracy were also calculated. Overfitting means that the model cannot fit well on datasets other than the training data, which is an indication of the model's poor generalizability. To reduce overfitting, all models were constructed with tenfold cross-validation. The diagnostic performance of the models was evaluated by using these indicators averaged over the tenfold cross-validation iterations. The generalizability was assessed by analyzing the AUC of each model in the internal and external validation sets.

### Statistical analysis

All statistical analyses and graphic production were performed using SPSS (v. 25; IBM), R (v. 4.11), and Python (v. 3.8.5). Normally distributed continuous variables are summarized as the means (± standard deviation), and non-normally distributed continuous variables are summarized as the medians (interquartile ranges). Continuous variables were analyzed by the Mann–Whitney *U* tests or independent sample *t* tests, and categorical variables were assessed by the Chi-square test or the Fisher’s exact tests. The DeLong test was used to compare the AUCs. A two-tailed *p* value of < 0.05 was considered significantly different.

## Results

### Patients

A total of 417 patients (mean age, 45.70 years; range, 11–95 years) with benign or borderline EOTs were recruited in this study. There were 309, 78, and 30 samples in the training set, internal validation set, and external validation set, respectively. No significant differences were seen in terms of age (*p* = 0.740), menopausal status (*p* = 0.581), parity (*p* = 0.219), abdominal symptoms (*p* = 0.362), CA125 (*p* = 0.118), HE4 (*p* = 1.000), and final diagnosis (*p* = 0.549) among these three datasets (Table 1). Comparing the benign and borderline lesion groups, two clinical characteristics (CA125 and HE4) and five radiological characteristics (ascites, maximum tumor diameter, tumor margins, SI of cystic component on FS T2W, and SI of solid component on FS T2W) were significantly different (*p* < 0.05) in the training set (Table 2). Other detailed information on the clinical and radiological characteristics of the enrolled patients is summarized in Tables 1 and 2, and Supplementary Materials (Additional file 1: Table S2).

**Table 1:** Clinical characteristics of training and validation sets

Cohort	Training set (*n* = 309)	Internal validation set (*n* = 78)	External validation set (*n* = 30)	*p* value
Age (years)	45.49 ± 16.06	46.94 ± 17.46	44.70 ± 19.17	0.740
Menopausal status				0.581
Postmenopausal	154 (49.8)	39 (50.0)	12 (40.0)	
Premenopausal	155 (50.2)	39 (50.0)	18 (60.0)	
Parity				0.219
Multipara	256 (82.8)	64 (82.1)	21 (70.0)	
Nullipara	53 (17.2)	14 (17.9)	9 (30.0)	
Abdominal symptoms				0.362
Pain and distention	135 (43.7)	37 (47.4)	17 (56.7)	
None	174 (56.3)	41 (52.6)	13 (43.3)	
CA125 (U/mL)				0.118
> 35	78 (25.2)	14 (17.9)	11 (36.7)	
≥ 35	231 (74.8)	64 (82.1)	19 (63.3)	
HE4^a^ (pmol/L)				1.000
Abnormal	20 (6.5)	5 (6.4)	2 (6.7)	
Normal	289 (93.5)	73 (93.6)	28 (93.3)	
Final diagnosis				0.549
Benign	224 (72.5)	57 (73.1)	19 (63.3)	
Borderline	85 (27.5)	21 (26.9)	11 (36.7)	
Histopathology				0.080
Serous	148 (47.9)	40 (51.3)	21 (70.0)	
Mucinous	141 (45.6)	33 (42.3)	6 (20.0)	
Others	20 (6.5)	5 (6.4)	3 (10.0)	

Data are presented as mean ± standard deviation for normally distributed continuous variables, median (interquartile range, IQR) for non-normally distributed continuous variables, or number (%) for categorical variables. HE4, human epididymis protein 4; CA125, carbohydrate antigen 125

^a^Normal value of HE4: postmenopausal woman < 121 pmol/L or premenopausal woman < 92.1 pmol/L

**Table 2:** Clinical and radiological characteristics for benign and borderline EOTs in training set

Cohort	Benign (*n* = 224)	Borderline (*n* = 85)	*p*
Age (years)	45.76 ± 16.10	44.78 ± 16.01	0.634
Menopausal status			0.266
Postmenopausal	116 (51.8)	38 (44.7)	
Premenopausal	108 (48.2)	47 (55.3)	
Parity			0.887
Multipara	186 (83.0)	70 (84.2)	
Nullipara	38 (17.0)	15 (17.6)	
Abdominal symptoms			0.321
Pain or distention	94 (42.0)	41 (48.2)	
None	130 (58.0)	44 (51.8)	
CA125 (U/mL)			0.000*
≥ 35	35 (15.6)	43 (50.6)	
< 35	189 (84.4)	42 (49.4)	
HE4^a^ (pmol/L)			0.001*
Abnormal	8 (3.6)	12 (14.1)	
Normal	216 (96.4)	73 (85.9)	
Ascites			0.000*
None	110 (49.1)	40 (47.1)	
Mild	98 (43.8)	29 (34.9)	
Moderate	15 (6.7)	7 (8.2)	
Massive	1 (0.4)	9 (10.6)	
Maximum tumor diameter (cm)	8.65 (6.22, 11.60)	10.40 (8.05, 13.65)	0.001*
Tumor margins			0.021*
Well-defined	223 (99.6)	81 (95.3)	
Ill-defined	1 (0.4)	4 (4.7)	
Number of loculi			0.107
Mild	136 (60.7)	43 (50.6)	
Multilocular	88 (39.3)	42 (49.4)	
SI of cystic on FS T2W			0.000*
Moderate	182 (81.3)	48 (56.5)	
Low	10 (4.5)	17 (20.0)	
High	5 (2.2)	6 (7.1)	
Mixed	27 (12.1)	14 (16.5)	
SI of solid on FS T2W			0.000*
Low	41 (18.3)	42 (49.4)	
High	0 (0.0)	1 (1.2)	
Mixed	12 (5.4)	10 (11.8)	
None	171 (76.3)	32 (37.6)	

Data are presented as mean ± standard deviation for normally distributed continuous variables, median (interquartile range, IQR) for non-normally distributed continuous variables, or number (%) for categorical variables. HE4, human epididymis protein 4; CA125, carbohydrate antigen 125; SI, signal intensity; FS: fat-suppressed; T2W: T2 weighted

*p < 0.05

^a^Normal value of HE4: postmenopausal woman < 121 pmol/L or premenopausal < 92.1 pmol/L

### Models building and evaluation

**Fig. 3:**
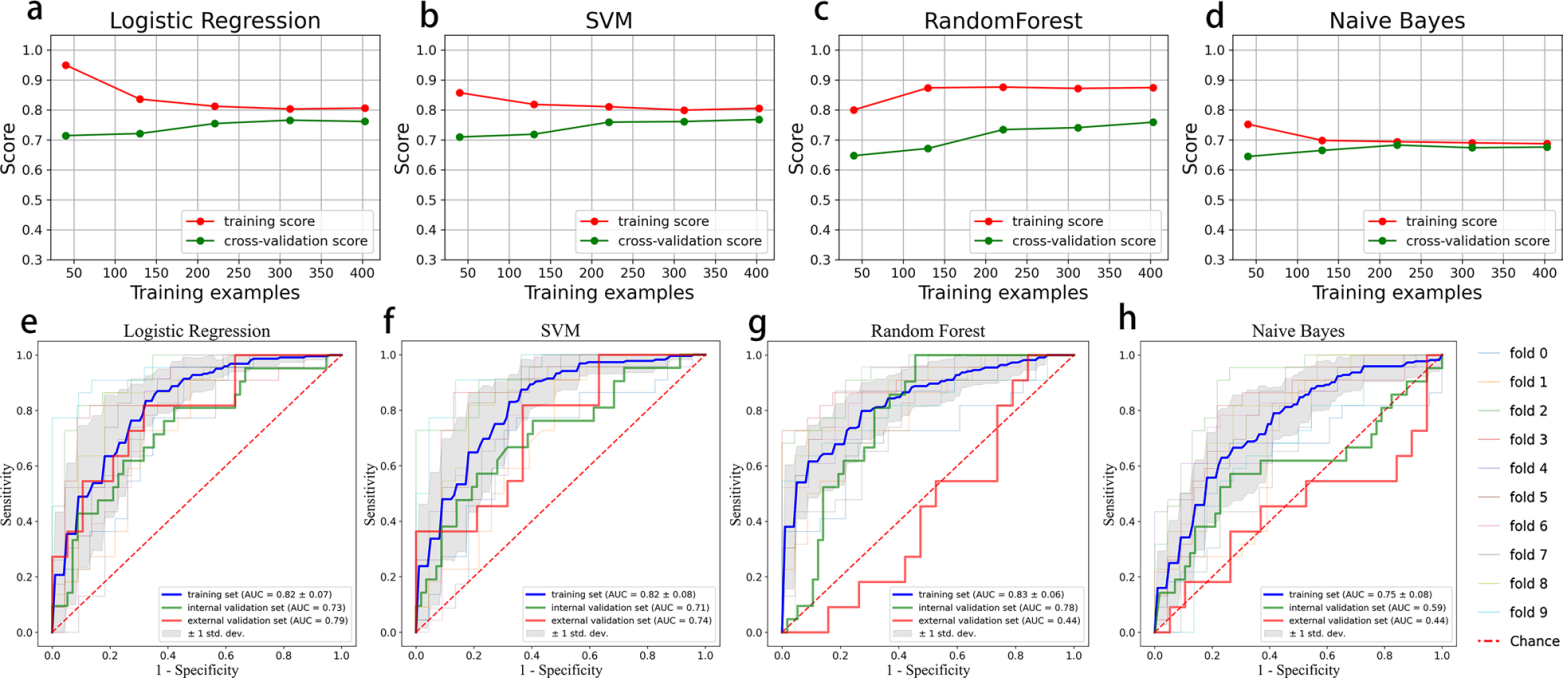
**a**–**d** The learning curves for four different radiomics models. The red and green curves represent the trend of the score with the increase in sample size in training and cross-validation data, respectively. The training and cross-validation scores of the logistic regression (LR) and SVM models were higher than those of the Naive Bayes (NB) model. The gap between the training and cross-validation scores of the LR or SVM models was smaller than that of the Random Forest (RF) model. **e**–**h** The receiver operating characteristic curves for four different radiomics models. Each light-colored curve represents each of the tenfold cross-validations (fold 0 to 9), and the dark blue curve represents their mean; the red and green curves represent internal and external validation sets, respectively. The LR model and SVM model had similar AUCs in the training set, but the LR model outperformed the SVM model in both the internal validation set and the external validation set. The RF model had the highest AUC in the training set but had low AUC in the external validation. The NB model had the lowest AUCs in all sets

According to the learning curves, the training scores of logistic regression and SVM models were around 0.8 and cross-validation scores were greater than 0.75 (Fig. 3a, b), which was superior to the Naive Bayes model (the training and cross-validation scores were all < 0.7, Fig. 3d). The gap between the training and cross-validation scores was lower in the logistic regression and SVM models (< 0.05) than that in the Random Forest model (> 0.10, Fig. 3c). According to the ROC curves, the LR model and SVM model had similar AUCs in the training set (AUC of 0.82 ± 0.07 and 0.82 ± 0.08, respectively). However, the LR model outperformed the SVM model in both the internal validation set (AUC of 0.73 vs. 0.71) and the external validation set (AUC of 0.79 vs. 0.74) (Fig. 3e, f). The RF model had an AUC of 0.83 ± 0.06 in the training set, but its AUC was only 0.44 in the external validation (Fig. 3g). The NB model had lowest AUCs in the training set (0.75 ± 0.08), internal validation set (0.59), and external validation set (0.44) (Fig. 3h). Therefore, the radiomics model constructed by the LR algorithm was optimal.

**Fig. 4:**
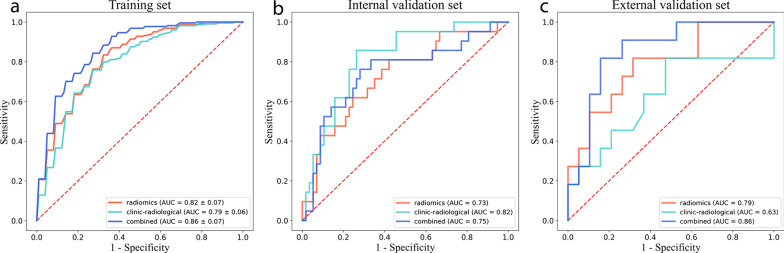
**a** Mean receiver operating characteristic (ROC) curves for the radiomics model, clinic-radiological model, and combined model over the tenfold cross-validation iterations. **b** ROC curves in the internal validation set. **c** ROC curves in the external validation set

After tenfold cross-validation, the combined model exhibited best diagnostic efficiency, with the AUC of 0.86 ± 0.07, specificity of 0.76 ± 0.11,sensitivity of 0.82 ± 0.13, PPV of 0.82 ± 0.11, and NPV of 0.78 ± 0.08 (Fig. 4a, Table 3). The radiomics model achieved a moderate AUC of 0.82 ± 0.07, which was still better than the performance of the clinical model (AUC of 0.79 ± 0.06). The ROC curves of the tenfold cross-validation and confusion matrix results of each model are presented in the Supplementary Materials (Additional file 1: Fig. S4–S6). There was no significant difference in AUCs among the three models in the internal validation set (Table 4). However, in the external validation set, the AUC value of the combined model was significantly better than that of the clinic-radiological model (0.86 vs. 0.63, *p* = 0.021 [DeLong test]). The comparison of diagnostic performance of the models in the validation sets is shown in Fig. 4b, c, and Table 4.

**Table 3:** Diagnostic performances of different models after tenfold cross-validation iterations

Models	Specificity	Sensitivity	PPV	NPV	Accuracy	AUC
Radiomics	0.71 ± 0.12	0.80 ± 0.09	0.79 ± 0.08	0.75 ± 0.08	0.76 ± 0.08	0.82 ± 0.07
Clinic-radiological	0.74 ± 0.08	0.71 ± 0.10	0.72 ± 0.06	0.74 ± 0.06	0.72 ± 0.05	0.79 ± 0.06
Combined	0.76 ± 0.11	0.82 ± 0.13	0.82 ± 0.11	0.78 ± 0.08	0.79 ± 0.08	0.86 ± 0.07

Values are mean (± standard deviation) over the cross-validation iterations

*PPV* positive predictive value, *NPV* negative predictive value, *AUC* area under the curve

**Table 4:** Comparison of models in internal and external validation sets

Models	Internal validation set			External validation set		
	Difference between areas	95% CI	*p* value	Difference between areas	95% CI	*p* value
Model1 versus Model2	0.088	− 0.034 to 0.210	0.159	0.160	− 0.156 to 0.476	0.320
Model1 versus Model3	0.018	− 0.044 to 0.079	0.574	0.067	− 0.105 to 0.239	0.446
Model2 versus Model3	0.070	− 0.032 to 0.172	0.178	0.227	0.034 to 0.421	0.021*

Model1: radiomics model; Model2: clinic-radiological model; Model3: combined model; CI, confidence interval

*p < 0.05

## Discussion

In this multicenter study, we investigated the feasibility of T2W MRI-based radiomics in differentiating between borderline and benign EOTs. After incorporating radiomics features, clinical, and conventional radiological characteristics, the combined model constructed by the LR algorithm had the best diagnostic performance in distinguishing borderline EOTs from benign EOTs. Generalizability was effectively demonstrated by showing consistently encouraging performance of the model when evaluated on the internal and external validation set.

Previous studies have shown that MRI-based radiomics can classify benign and malignant ovarian tumors with high AUCs of around 0.90 [16, 21]. However, one limitation shared in these ﻿mentioned studies is the inclusion of borderline EOTs in the category of ovarian malignancies. Within the clinical scenario, borderline EOTs should be regarded as a separate category since their treatment differs from both benign and malignant EOTs [10, 11, 35]. Additionally, since the number of patients with borderline EOTs recruited in studies is often small, the diagnostic performance of the radiomics model may not obviously change, even if many borderline EOTs are misdiagnosed as benign. For comparison, we not only focused on borderline EOTs but also applied the SMOTE algorithm to oversample the minority class (borderline EOTs) to reduce the adverse effect caused by sample imbalance [34].

Compared with one prior study, where a radiomics model based on the dynamic contrast-enhanced (DCE)-MRI can achieve good performance in diagnosing ovarian tumors, with the AUC of more than 0.86 [22], we used T2W MRI to construct models and obtained similar encouraging diagnostic performance in differentiating between benign and borderline EOTs. Although radiomics using non-contrast MRI has been less well studied in ovarian tumors, this approach has shown promising results in the diagnosis of liver and breast tumors [36, 37]. Recently, one study showed that radiomics based on the FS T2W images could help clinicians differentiate borderline EOTs from malignancies (accuracy of 0.99 from the three-dimensional model) [38]. Similar to the mentioned study, we only applied FS T2W images for modeling, thus providing additional evidence for the application of T2W MRI-based radiomics in borderline EOTs.

Early diagnosis of borderline EOTs is crucial for clinical decision making because patients with borderline EOTs required stricter preoperative evaluation and postoperative follow-up. If a precise preoperative diagnosis is achievable, restaging surgery can be avoided. However, there is currently no easy and reliable means of screening. O-RADS MRI recommended morphological sequences (T2, T1 with and without fat suppression, and T1 after gadolinium injection) and functional sequences (perfusion and diffusion‐weighted sequences) for every patient [14]. However, high costs, motion artifacts, and too long scanning time restrict its widespread application in screening. Our results showed that the model constructed by the T2W sequence alone could effectively differentiate between benign and borderline EOTs preoperatively, thus providing a new idea for screening high-risk women with ovarian tumors.

In this study, we also compared the performance of the combined model with that of the clinic-radiological model. The diagnostic performance in the combined model was superior to that in the clinic-radiological model, especially when evaluated on the external validation set. Therefore, the combination of radiomics features and clinical and conventional radiological characteristics may have the potential to improve the diagnostic performance and generalizability of the model. The combined model in our study achieved better performance than that of the model combining ultrasound and clinical features in a recent study (AUC of 0.86 vs. 0.825) [39]. Additionally, in clinical application, misdiagnosis of borderline ovarian tumors should be avoided because it will lead to the risk of recurrence and deterioration, thus requiring the model to have high sensitivity. Compared with the model with a sensitivity of < 0.75 [39], the combined model in our study had an increased sensitivity of 0.82, indicating that it may have the potential to address this need for the detection of borderline EOTs.

There are several novelties and strengths in the methods of this study. First, as suggested in the Radiomics Quality Score (RQS) [19], we recruited patients from multiple centers and performed internal and external validation, which contributes to the robustness of our results. Second, one of the main limitations of most radiomics studies is the homogeneity of the data, which adversely impacts the generalization of results. To overcome this deficiency, our study included data from seven MRI scanners regardless of manufacturer, protocol, and field strength, and the ComBat harmonization algorithm minimized the bias caused by different MRI scanners. This algorithm has been confirmed not only to reduce the batch effects caused by different imaging protocols [32, 33] but also to partially improve the predictive performance of the radiomics model [40]. Furthermore, different machine learning algorithms may perform differently on the same data since they have disparate mathematical principles. Therefore, we used four different machine learning algorithms for modeling and compared the differences in the algorithms’ fitting effect and generalization performance. This increased the reliability of our results by reducing the bias caused by choosing a single machine learning algorithm.

Nevertheless, there are several limitations of this study. First, the amount of data in the external validation set used in this study was small. This is due to the difficulty of medical image acquisition and our desire to approximate the actual data distribution with as large a training sample as possible. Second, potential selection bias may exist in this study because of the retrospective study design. Thus, prospective multicenter validation of a large sample size is needed in our further study. Third, this study did not compare the performance between T2W and other sequences. Therefore, further study is required to evaluate the performance of radiomics based on multiparametric MRI.

## Conclusion

In conclusion, the radiomics based on T2W MRI had the potential to differentiate between benign and borderline EOTs effectively. Our findings could offer potential guidance for preoperative clinical decision making and merit further evaluations and development for future clinical applications.

## Supplementary Information

**Additional file 1:** Supplementary materials on centers information, radiomics features, ComBat harmonization, feature selection, additional tables, and additional figures.

## Data Availability

The datasets generated and/or analyzed during the current study are not publicly available because the subjects did not provide written consent for their data to be publicly shared.

## Abbreviations

CA125: Carbohydrate antigen 125
DCE: Dynamic contrast-enhanced
EOT: Epithelial ovarian tumor
FIGO: International Federation of Gynecology and Obstetrics
FS: Fat-suppressed
HE4: Human epididymis protein 4
IBSI: Image biomarker standardization initiative
ICC: Intraclass correlation coefficient
LASSO: Least Absolute Shrinkage and Selection Operator
LR: Logistic regression
NB: Naive Bayes
RF: Random Forest
RQS: Radiomics Quality Score
SI: Signal intensity
SMOTE: Synthetic minority oversampling technique
SVM: Support vector machine
T2W: T2-weighted
VOI: Volume of interest
