# Supplementary material for: T2-weighted MRI-based radiomics for discriminating between benign and borderline epithelial ovarian tumors: a multicenter study
Source: Insights Imaging. 2022 Aug 9;13:130. doi: 10.1186/s13244-022-01264-x (PMC9363551; doi:10.1186/s13244-022-01264-x)
Supplement: Supplementary file 1 — Additional file 1: Supplementary materials on centers information, radiomics features, ComBat harmonization, feature selection, additional tables, and additional figures. [file 13244_2022_1264_MOESM1_ESM.pdf]

ELECTRONIC SUPPLEMENTARY MATERIAL

T2-weighted MRI-Based Radiomics for Discriminating Between Benign and Borderline Epithelial Ovarian Tumors: A Multicenter Study

Contents

Section 1: Details of centers 2

Section 2: Custom settings and detailed information on radiomics features

.... 2

Section 3: ComBat harmonization 2

Section 4: Feature selection.. 3

Section 5: Tables 4

Section 6: Figures 8

Section 1: Details of centers

The multicenter study was conducted jointly by 3 centers:

Center I: the Affiliated Suzhou Hospital of Nanjing Medical University, Suzhou, China (1.5/3.0 T, Aera/ Skyra, Siemens, Erlangen, Germany; 1.5 T, Achieva, Philip, Netherlands).

Center II: the Affiliated Huaian No. 1 People's Hospital of Nanjing Medical University, Huaian, China (1.5/3.0 T, Aera/ Avanto/ Verio/ Spectra, Siemens, Erlangen, Germany).

Center III: the First Affiliated Hospital of Soochow University, Suzhou, China (3.0 T, Skyra, Siemens, Erlangen, Germany; 3.0 T, Ingenia, Philip, Netherlands; 3.0 T, Signa, GE, Milwaukee WI, USA).

Section 2: Custom settings and detailed information on radiomics features

Versions of Python and packages: Python v3.8.8**,** PyRadiomics v3.0.1**,** Numpy v1.19.2**,** SimpleITK v2.1.0**,** PyWavelet v1.1.1
Configuration Settings of PyRadiomics: "normalize": True, "normalizeScale": 100, "resampledPixelSpacing": [3, 3, 3], "interpolator": sitkBSpline, "binWidth": 5, "voxelArrayShift": 300, "LoG": sigma: [3.0, 4.0, 5.0]

Parameters were set according to the example file provided on GitHub (https://github.com/Radiomics/pyradiomics/blob/master/examples/exampleSettings/exampleMR_5mm.yaml). A total of 1130 radiomics features were extracted in this study and can be divided into 5 groups: (I) 18 first-order features; (II) 14 shape features; (III) 75 second-order features, including 24 gray level co-occurrence matrix features, 16 gray level run length matrix features, 16 gray level size zone matrix features, 14 gray level dependence matrix features, and 5 neighborhood gray-tone difference matrix features; (IV) 279 features derived from the Laplacian-of-Gaussian filter; and (V) 744 features derived from the wavelet filter. A detailed description of the various features and can be found on the website of PyRadiomics (https://pyradiomics.readthedocs.io/en/latest/features.html).

Section 3: ComBat harmonization

The ComBat harmonization algorithm was originally proposed for the genetics field to address the batch effects seen in microarray analysis. The ComBat harmonization

method is derived from the location and scales family of corrections where it is assumed that the error introduced by the batch differences can be corrected by standardizing the means and variances across the batches. The method directly applies to the radiomics feature values and estimates the scanner effect by matching the statistical distributions of the feature values measured in ROI *j* (sample) for each scanner *i* (batch)[1]:

$$\boldsymbol{Y}_{\mathbf{ij}} = \boldsymbol{\alpha} + \boldsymbol{X}_{\boldsymbol{ij}}\boldsymbol{\beta} + \boldsymbol{\gamma}_{\mathbf{i}} + \boldsymbol{\delta}_{\mathbf{i}}\boldsymbol{\varepsilon}_{\mathbf{ij}}$$

where α is the average value for feature, *X* is the design matrix for the covariates of interest, β is the vector of regression coefficients corresponding to each covariate, γ_i is an additive batch effect, and δ_i is a multiplicative batch effect and ε_{ij} is an error. By estimating the additive and multiplicative batch effect, the corrected values are obtained using:

$$\boldsymbol{Y}_{\boldsymbol{ij}}^{\boldsymbol{Combat}} = \frac{\boldsymbol{Y}_{\mathbf{ij}} - \widehat{\boldsymbol{\alpha}} - \boldsymbol{X}_{\boldsymbol{ij}}\widehat{\boldsymbol{\beta}} - \widehat{\boldsymbol{\gamma}_{\iota}}}{\widehat{\boldsymbol{\delta}_{\iota}}} + \widehat{\boldsymbol{\alpha}}$$

Where $\hat{\alpha}$,$\hat{\beta}$,$\widehat{\gamma_{\iota}}$ and $\widehat{\delta_{\iota}}$ are estimators of α, β, γ_i,and δ_i, respectively.

Different types of MRI scanners and different field intensities might influence radiomics features, which is similar to the problem called batch effects in genomics. In this study, different batches were considered different MR scanners, and no biologic covariate was used.

[1] Orlhac F, Lecler A, Savatovski J et al (2020) How can we combat multicenter variability in MR radiomics? Validation of a correction procedure. Eur Radiol. 10.1007/s00330-020-07284-9

Section 4: Feature selection

A total of 1130 radiomics features were extracted. After ICC analysis (1002/1130), Mann–Whitney *U* test (705/1002), Spearman correlation analysis (112/705), and Least Absolute Shrinkage and Selection Operator algorithm, 33 features were finally retained.

Section 5: Tables

Table S1 Different scanners and imaging parameters of FS T2W used in the study

Manufacturer	Type	number	Sequence	TR	TE	Magnetic Field	Slice Thickness	FOV
SIEMENS	Aera	134	SE	4410 - 5250	85 - 87	1.5	5 - 10	640*640/320*320
SIEMENS	Avanto	80	SE	1100 - 5415	94 - 121	1.5	5 - 9	384*512
SIEMENS	Verio	66	SE	1700 - 5000	97 - 100	3.0	5 - 10	320*320
SIEMENS	Spectra	54	SE	1600 - 4500	90 - 96	3.0	4.5 - 7	240*320/640*640
SIEMENS	Skyra	26	SE	3020 - 5000	76 - 101	3.0	5 - 10	320*320/640*640
Philips	Achieva	35	SE	4000 - 5332	70	1.5	5.7 – 10.5	480*480
Philips	Ingenia	18	SE	3586	80	3.0	6 - 9	960*960
GE	Signa	4	SE	3960 - 4881	88 - 105	3.0	4 - 10	512*512

TR: repetition time; TE: echo time; SE: spin-echo; FOV: field of view

Table S2 clinical and radiological characteristics in validation sets

Cohort	Internal validation set			External validation set		
	Benign (n = 57)	Borderline (n = 21)	*P*	Benign (n = 19)	Borderline (n = 11)	*P*
Age (years)	48.60 ± 16.99	42.48 ± 18.35	0.171	47.74 ±21.60	39.45 ± 13.52	0.261
Menopausal status			0.799			0.442
postmenopausal	29 (50.9)	10 (47.6)		10 (52.6)	8 (72.7)	
premenopausal	28 (49.1)	11 (52.4)		9 (47.4)	3 (27.3)	
Parity			0.627			0.419
multipara	48 (84.2)	16 (76.2)		12 (63.2)	9 (81.8)	
nullipara	9 (15.8)	5 (23.8)		7 (36.8)	2 (18.2)	
Abdominal symptoms			0.297			0.132
pain or distention	25 (43.9)	12 (57.1)		13 (68.4)	4 (36.4)	
none	32 (56.1)	9 (42.9)		6 (31.6)	7 (63.6)	
CA125 (U/ml)			0.000*			0.004*
≥35	3 (5.3)	11 (52.4)		3 (15.8)	8 (72.7)	
<35	54 (94.7)	10 (47.6)		16 (84.2)	3 (27.3)	
HE4[a] (pmol/L))			0.025*			1.000
abnormal	1 (1.8)	4 (19.0)		1 (5.3)	1 (9.1)	
normal	56 (98.2)	17 (81.0)		18 (94.7)	10 (90.9)	
Ascites			0.020*			0.644
none	33 (57.9)	6 (28.6)		9 (47.4)	3 (27.3)	
mild	23 (40.4)	12 (57.1)		5 (26.3)	4 (36.4)	
moderate	1 (1.8)	3 (14.3)		4 (21.1)	4 (36.4)	
massive	0 (0.0)	0 (0.0)		1 (5.3)	0 (0.0)	
Maximum tumor diameter (cm)	8.4 (6.45, 11.90)	13.13 ± 6.41	0.023*	11.40 (7.40, 13.70)	7.40 (5.40, 15.40)	0.451
Tumor margins			-			-
well-defined	57 (100.0)	21 (100.0)		19 (100.0)	11 (100.0)	
ill-defined	0 (0.0)	0 (0.0)		0 (0.0)	0 (0.0)	
Number of loculi			0.010*			0.466
mild	35 (61.4)	6 (28.6)		9 (47.4)	7 (63.6)	
multilocular	22 (38.6)	15 (71.4)		10 (52.6)	4 (36.4)	
SI of cystic on FS T2W			0.000*			0.845
moderate	49 (86.0)	8 (38.1)		13 (68.4)	6 (54.4)	
low	0 (0.0)	4 (19.0)		1 (5.3)	1 (9.1)	
high	4 (7.0)	3 (14.3)		0 (0.0)	0 (0.0)	
mixed	4 (7.0)	6 (28.6)		5 (26.3)	4 (36.4)	

SI of solid on FS T2W			0.051			0.349
low	14 (24.6)	9 (42.9)		5 (26.3)	4 (36.4)	
high	0 (0.0)	0 (0.0)		0 (0.0)	0 (0.0)	
mixed	1 (1.8)	2 (9.5)		1 (5.3)	2 (18.2)	
none	42 (73.7)	10 (47.6)		13 (68.4)	5 (45.5)	

Data are presented as mean ± standard deviation for normally distributed continuous variables, median (interquartile range, IQR) for non-normally distributed continuous variables, or number (%) for categorical variables. HE4, human epididymis protein 4; CA125, carbohydrate antigen 125; SI, signal intensity; FS: fat-suppressed; T2W: T2 weighted.

[a]: normal value of HE4: postmenopausal woman < 121 pmol/L or premenopausal woman < 92.1 pmol/L, *: $P < 0.05$.

Table S3 Number of correct diagnoses for different histopathologic subtypes by different models in internal and external validation sets.

Model	Internal validation set			External validation set		
	Serous (n = 40)	Mucinous (n = 33)	Others (n = 5)	Serous (n = 21)	Mucinous (n = 6)	Others (n = 3)
Radiomics	31	20	2	16	3	2
Clinic-radiological	29	26	5	16	1	1
Combined	33	20	4	20	2	1

Section 6: Figures

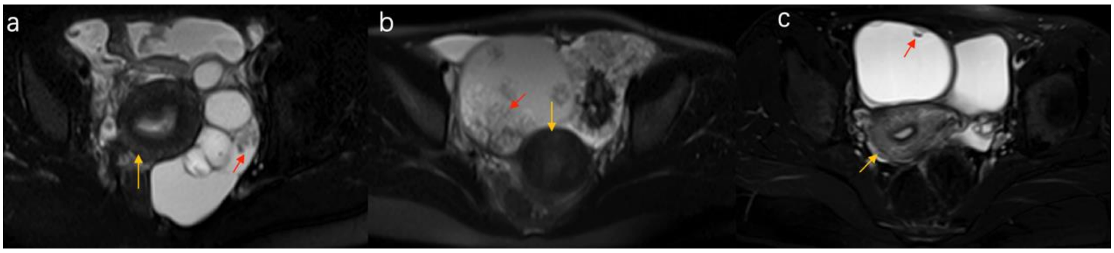

Fig S1 (a) A 38-year-old woman with a left borderline serous cystadenoma who had high signal intensity of the solid component (red arrow) compared with adjacent external myometrium (yellow arrow); (b) A 32-year-old woman with a right borderline serous cystadenoma who had mixed signal intensity of the solid component (red arrow) compared with adjacent external myometrium (yellow arrow); (b) A 25-year-old woman with a right benign serous cystadenoma who had low signal intensity of the solid component (red arrow) compared with adjacent external myometrium (yellow arrow).

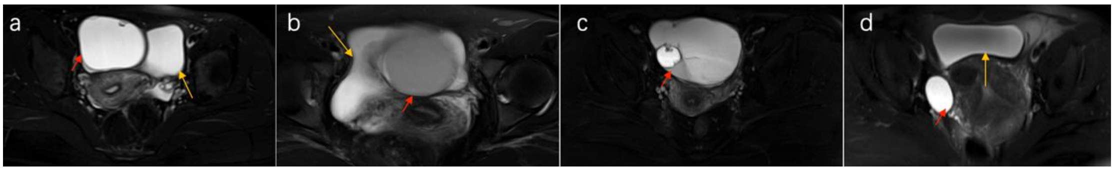

Fig S2 (a) A 25-year-old woman with a right benign serous cystadenoma who had moderate signal intensity of the cystic component (red arrow) compared with urinary bladder (yellow arrow); (b) A 37-year-old woman with a right borderline mucinous cystadenoma who had low signal intensity of the cystic component (red arrow) compared with urinary bladder (yellow arrow); (c) A 30-year-old woman with a left benign mucinous cystadenoma who had mixed signal intensity of the cystic component (red arrow); (d) A 43-year-old woman with a right borderline mucinous cystadenoma who had high signal intensity of the cystic component (red arrow) compared with urinary bladder (yellow arrow).

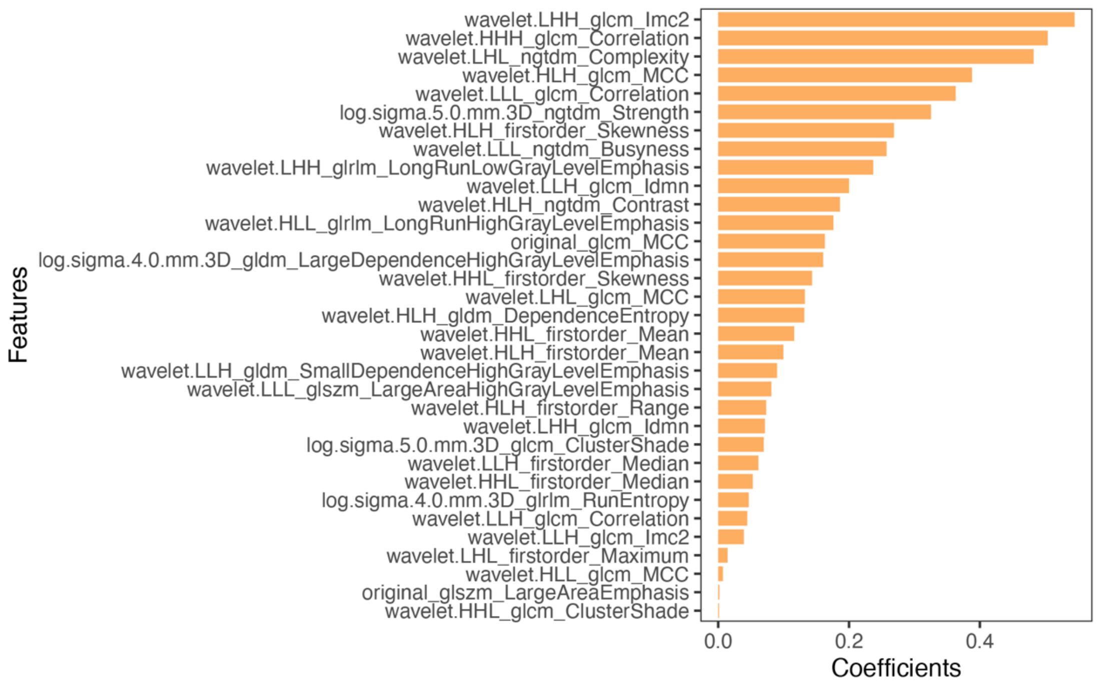

Fig. S3 the remaining radiomics features after feature selection. The y-axis shows the selected 33 radiomics, and the x-axis shows the coefficient of each feature after the Least Absolute Shrinkage and Selection Operator algorithm.

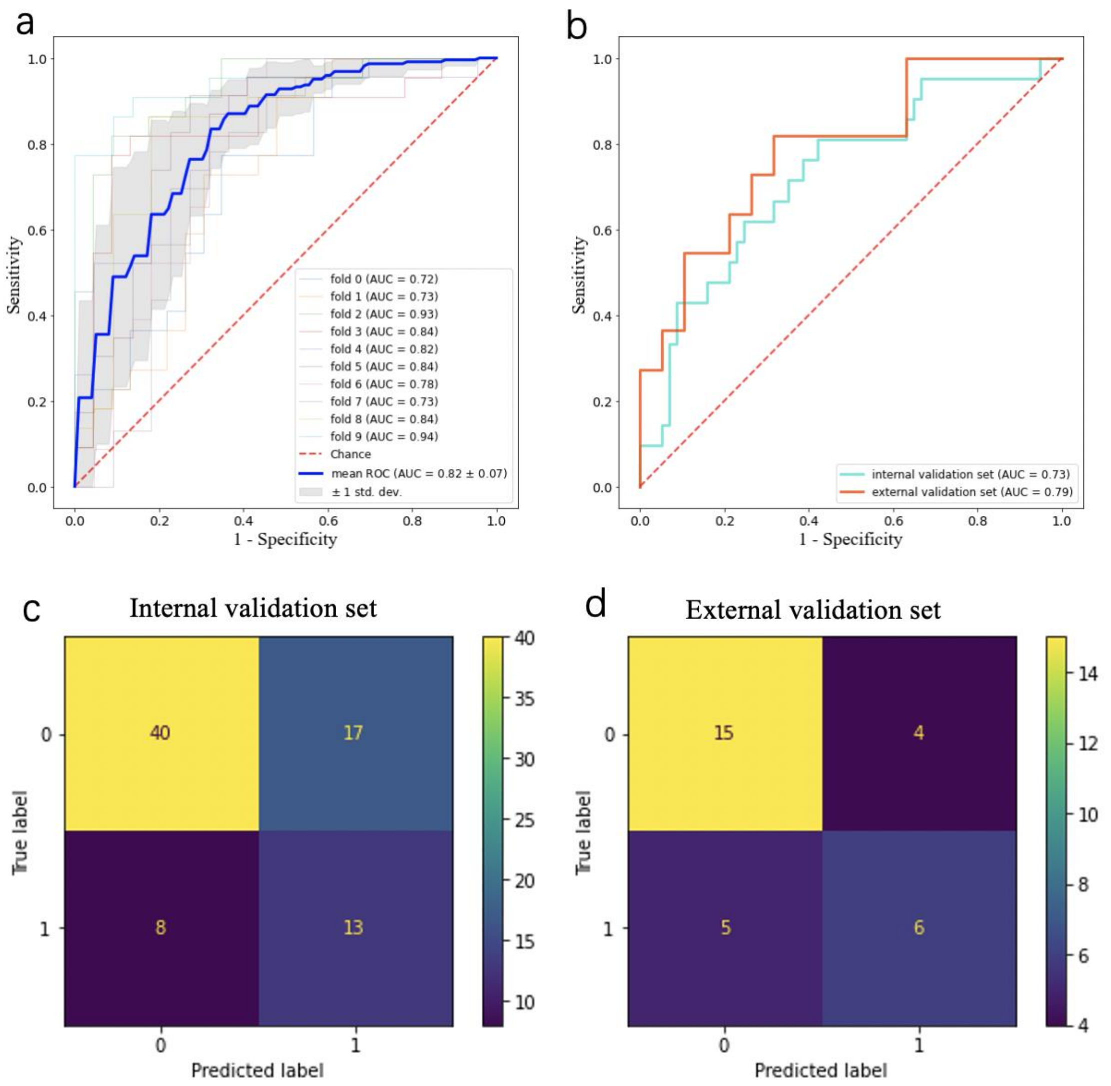

Fig. S4 The detailed performance of the radiomics model. (a) Mean and tenfold cross-validation ROC curves for the model in discriminating between benign and borderline EOTs. (b) ROC curves in internal and external validation sets. (c) the confusion matrix in the internal validation set. (d) the confusion matrix in the external validation set. “0”, benign EOTs; “1”, borderline EOTs.

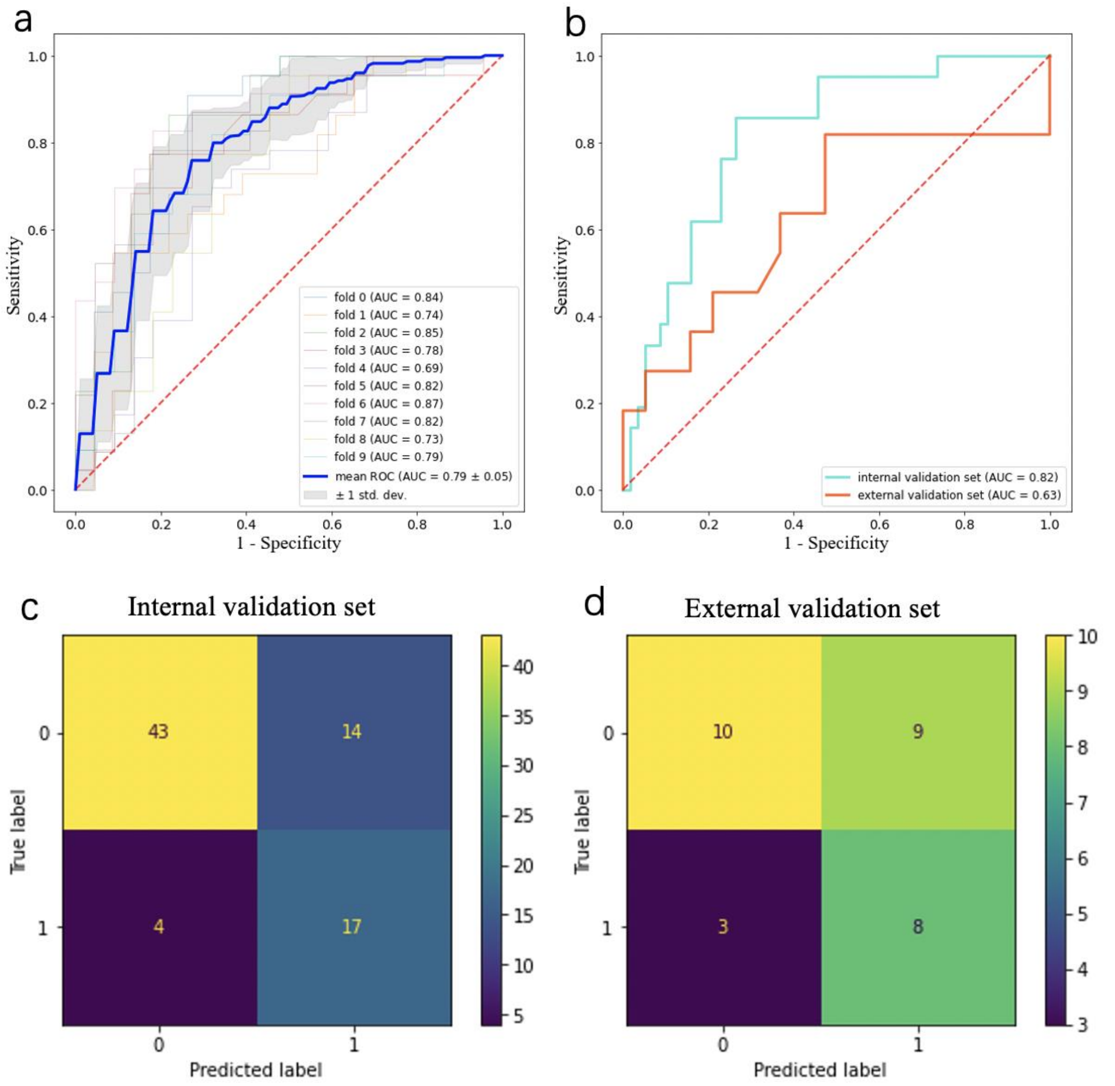

Fig. S5 The detailed performance of the clinic-radiological model. (a) Mean and tenfold cross-validation ROC curves for the model in discriminating between benign and borderline EOTs. (b) ROC curves in internal and external validation sets. (c) the confusion matrix in the internal validation set. (d) the confusion matrix in the external validation set.

“0”, benign EOTs; “1”, borderline EOTs.

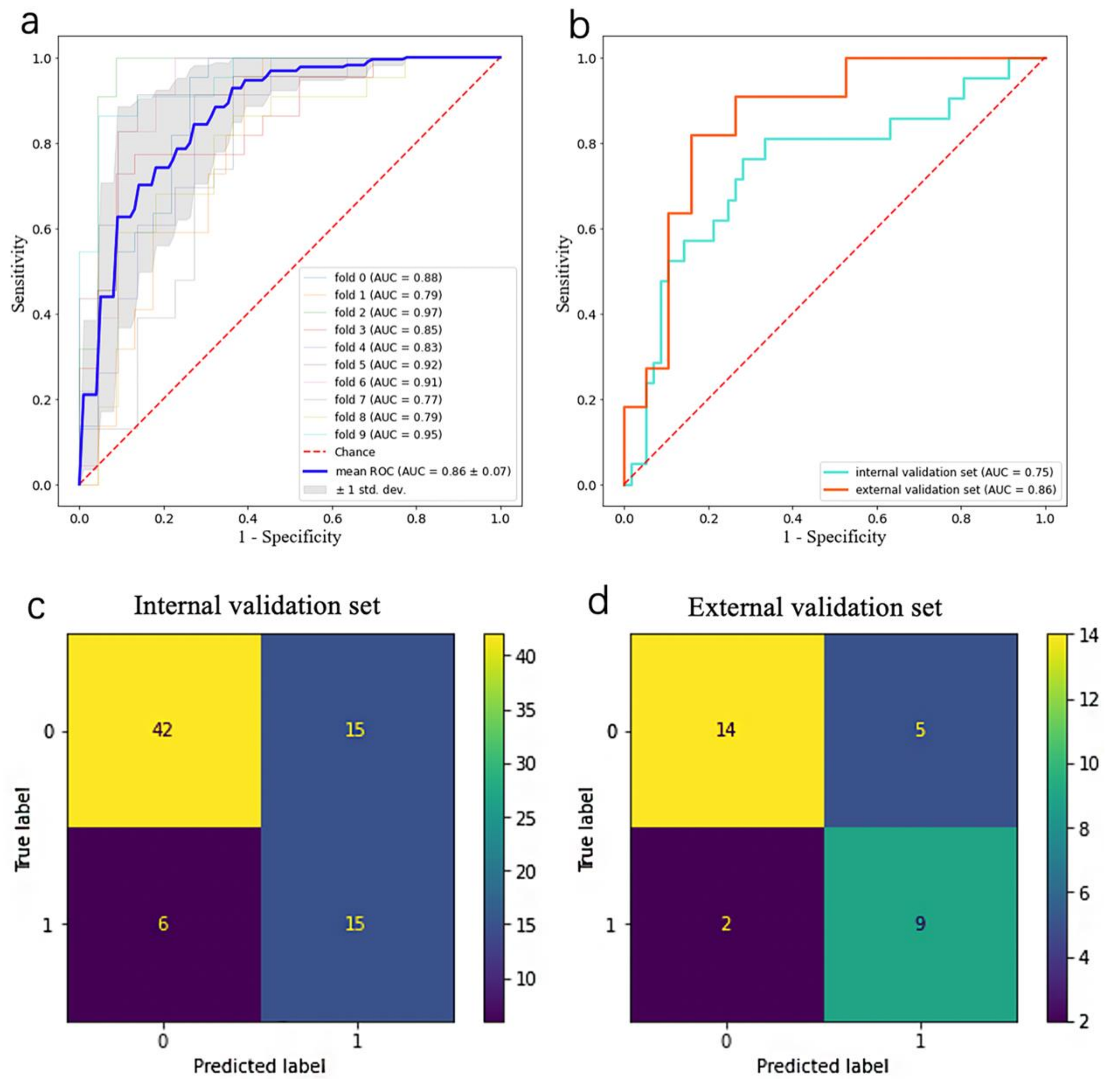

Fig. S6 The detailed performance of the combined model. (a) Mean and tenfold cross-validation ROC curves for the model in discriminating between benign and borderline EOTs. (b) ROC curves in internal and external validation sets. (c) the confusion matrix in the internal validation set. (d) the confusion matrix in the external validation set. “0”, benign EOTs; “1”, borderline EOTs.
